# Compliance-free, analog RRAM devices based on SnO_x_

**DOI:** 10.1038/s41598-024-64662-9

**Published:** 2024-06-19

**Authors:** Suresh Kumar Garlapati, Firman Mangasa Simanjuntak, Spyros Stathopoulos, Syed Jalaluddeen A, Mari Napari, Themis Prodromakis

**Affiliations:** 1 Department of Materials Science and Metallurgical Engineering, Indian Institute of Technology Hyderabad, Hyderabad, 502285 India; 2 School of Electronics & Computer Science, University of Southampton, Southampton, SO17 1BJ UK; 3 Centre for Electronics Frontiers, School of Engineering, University of Edinburgh, Edinburgh, UK; 4 Department of Physics, King’s College London, London, WC2R 2LS UK

**Keywords:** Engineering, Materials science, Nanoscience and technology, Physics

## Abstract

Brain-inspired resistive random-access memory (RRAM) technology is anticipated to outperform conventional flash memory technology due to its performance, high aerial density, low power consumption, and cost. For RRAM devices, metal oxides are exceedingly investigated as resistive switching (RS) materials. Among different oxides, tin oxide (SnO_x_) received minimal attention, although it possesses excellent electronic properties. Herein, we demonstrate compliance-free, analog resistive switching behavior with several stable states in Ti/Pt/SnO_x_/Pt RRAM devices. The compliance-free nature might be due to the high internal resistance of SnO_x_ films. The resistance of the films was modulated by varying Ar/O_2_ ratio during the sputtering process. The I–V characteristics revealed a well-expressed high resistance state (HRS) and low resistance states (LRS) with bipolar memristive switching mechanism. By varying the pulse amplitude and width, different resistance states have been achieved, indicating the analog switching characteristics of the device. Furthermore, the devices show excellent retention for eleven states over 1000 s with an endurance of > 100 cycles.

## Introduction

The need to address the scaling limitations of DRAM and Flash memories shifted the attention to promising alternative technologies such as resistive random access memory (RRAM), ferroelectric random access memory (FRAM), magnetoresistive random access memory (MRAM), and phase-change memory (PCM)^1–4^. Among the aforementioned non-volatile memory (NVM) technologies, the low power consumption, sub-nanoseconds switching speed, and small feature size that allows for the highest possible planar array density (4F^2^) enables RRAM to be distinct compared to the others^4–6^. Furthermore, its acclaimed characteristics such as high device density, high endurance of 10^12^ cycles, and long retention times (10 years at 85 °C) boosted its popularity in the non-volatile memory (NVM) market tremendously^4,7–10^. Recent growth in interest for artificial intelligence (AI) mandated systems with low latency in data transfer between the memory and the computation (edge computing) as a requirement to provide real-time information^11^. This can be achieved by simulating biological synapses with parallel computing (Neuromorphic computing)^12,13^. However, the conventional systems with Von Neumann architecture severely bottleneck the progress as the memory, and the processing unit are separate and have high latency in the movement of the data. On the other hand, RRAM demonstrates non-volatile computing-in-memory (nvCIM), multistate operation characteristics, and capability of building neural network,making it a suitable candidate for neuromorphic computing applications^11,13–16^.

For RRAM devices, binary metal oxides are perfect materials due to their exceptional characteristics, such as structural simplicity, a wide variety of fabrication and processing routes, and the inevitable existence of non-stoichiometry in their structure. Especially, the transition metal oxides (TMO) such as TiO_x_, NiO, ZnO, HfO_2_ and TaO_x_ have gained significant attention for RRAM applications due to their simple fabrication processes and high compatibility with Si technologies^17–22^. Among these oxides, TiO_x_ remains an extensively investigated switching material due to its high dielectric constant (86–170), thermal stability, and wide bandgap (3–3.2 eV), which reduces leakage current^23^. Besides TiO_x_, dielectric metal oxides such as HfO_2_ and TaO_x_ have also been gaining attention due to their excellent switching properties, high compatibility with CMOS technologies, self-compliance, and self-rectifying behaviour, benefiting crossbar arrays^21,22,24,25^. However, semiconducting metal oxide such as SnO_x_ received lesser attention, albeit it is abundant, low cost, exhibits excellent electronic transport properties and can be employed as transparent conducting oxide^26–28^. Additionally, the n-type semiconducting nature of SnO_x_ attributed to the oxygen vacancies and its displacement resistance property (lower chance of single or multi-bit event upset) due to its high Frenkel defect energy (7 eV) can make tin-based oxide highly sought-out switching material for RRAM devices^10,28^.

RRAM devices based on tin oxide have been reported in the literature. Kazuki et al. reported unipolar RS phenomena for Pt/SnO_2_/Pt structure with good endurance (100 cycles) and retention (10^4^ s) properties^29^. On the other hand, Jin et al. reported bipolar RS characteristics for on Al/SnO_x_/Pt structures, where different annealing conditions involving nitrogen atmosphere at 300 °C have been used for SnO_x_. Pan et al. have observed synaptic plasticity in solution processed SnO_2_ devices by controlling the oxygen vacancy migration to modify the Schottky barrier at the Au/SnO_2_ interface. Phuong et al. reported forming-free RS mechanism with individual conductance states for different input sequences suggesting potential artificial synapse applications. However, the retention and endurance characteristics for the states were absent in all of these reports^10,30,31^. Chih-Chieh et al. and Chandra et al. demonstrated top electrode-free SnO_x_ RRAM devices. The latter reported endurance cycles of 500, very low switching voltages and a resistive window of 15 and the former reported the same endurance cycles with solution processed SnO_x_ film^32,33^. Electrochemical metallization (ECM) RS was also reported in SnO_x_ based devices. Yun et al. (Ag/SnO_2_/Pt) reported that microwave treatment influences the diameter of the filament, which in turn increases the formation and rupture times of the filament for improved stability. Chen et al. investigated RS behaviour for Ag/SnO_x_/ITO structure to observe the impact of annealing on the ratio of the resistances states, as it affects the memory performance^34,35^. However, SnO_x_ based RRAM devices operating compliance-free and exhibiting stable multistate operation are very scarcely reported. Herein, we report RRAM devices with SnO_x_ switching layer that exhibits analog switching behaviour and operates without any compliance current (I_cc_). The devices showed 11 stable resistance states with high retention and endurance values confirming the prospect of SnO_x_ for neuromorphic computing applications.

## Experimental work

RRAM devices were fabricated on thermally oxidized (200 nm SiO_2_) 6″ Si wafers. All layers (bottom electrodes, oxide layer, top electrodes) of the devices were patterned by photolithography process. The steps involved in patterning process are spin coating 4 µm thick AZ2070 negative photoresist, pre-exposure baking at 110 °C for 1 min, exposing (16 s) to 365 nm ultra-violet (UV) light using EVG 620 TB mask aligner. Next, post-exposure baking at 110 °C for 1 min, developing using AZ726 MIF for 90 s, depositing required layers, followed by lift-off in NMP (1-Methyl-2-pyrrolidone) solvent. Bottom electrodes, Ti(10 nm)/Pt(15 nm), were deposited by using an e-beam evaporator (Leybold Lab 700), 30 nm SnO_x_ was deposited by using a magnetron sputtering (Angstrom Engineering) machine. Top electrodes (15 nm of Pt) were deposited using the same e-beam evaporator. SnO_x_ films were deposited using the reactive sputtering method with a 4″ Sn target (Testbourne Ltd., UK) with 1:2 mass flow ratio of argon and oxygen at 1 mTorr pressure. The films were deposited at a power of 70 W.

Structural characterization of as-deposited SnO_x_ thin films has been carried out using X-ray diffraction (XRD, Rigaku SmartLab) and X-ray photoelectron spectroscopy (XPS) techniques. Since the films are very thin (30 nm), a grazing incidence angle of 1° has been used and scanned from 20° to 60° with a step size of 0.002° for XRD studies. To investigate the SnOx stoichiometry and oxygen vacancy concentration at the top electrode interface, SnOx films with a 5 nm Pt layer were analyzed by XPS using a Thermo Scientific Theta Probe spectrometer equipped with a monochromatic Al-Kα (1.4866 keV) x-ray source. The data were analyzed using CasaXPS software for peak modeling by applying Shirley background and Gaussian–Lorentzian line shape (GL (30)). The spin–orbit coupling ratio of 3:2 was used as a constraint in the fitting of the Sn 3d_5/2_ and 3d_3/2_ peaks. The binding energies were adjusted to the adventitious carbon peak (284.8 eV) at the sample surface. To obtain the depth information the sample was etched by 2 keV, 1uA Ar + ion beam sputtering in 30 s intervals.

Next, electrical characterization of prepared RRAM devices has been performed at ambient conditions (room temperature, atmospheric pressure, and in the air) on 5 × 5 µm^2^ area devices using our ARC ONETM31 measurement system and Cascade SUMMIT 12000B probe station. The current (I)- voltage (V) measurements have been carried out by applying voltage bias and ground potential at the top and bottom electrodes, respectively. The read voltage and pulse widths are 0.2 V and 10 ms, respectively.

## Results and discussion

**Figure 1:**
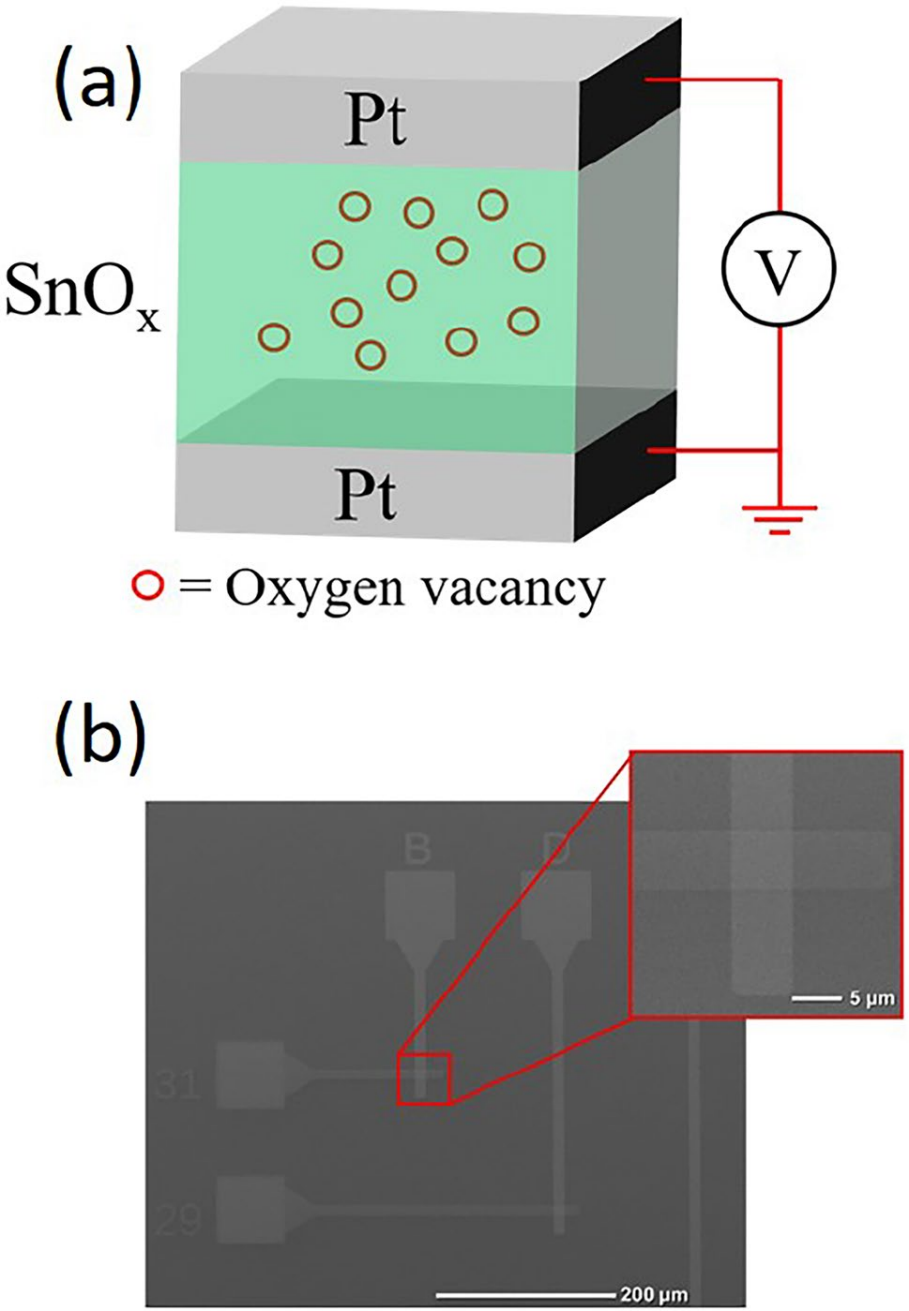
(**a**) The schematic shows Pt/SnO_x_/Pt RRAM devices. (**b**) The SEM image shows the structure of SnO_x_ RRAM devices.

SnO_x_ based RRAM devices have been prepared using sputtering with Ti/Pt and Pt as bottom and top electrodes, respectively. The schematic of the RRAM device is shown in Fig. 1a. The corresponding scanning electron microscopy (SEM) image of the devices is also shown in Fig. 1b.

**Figure 2:**
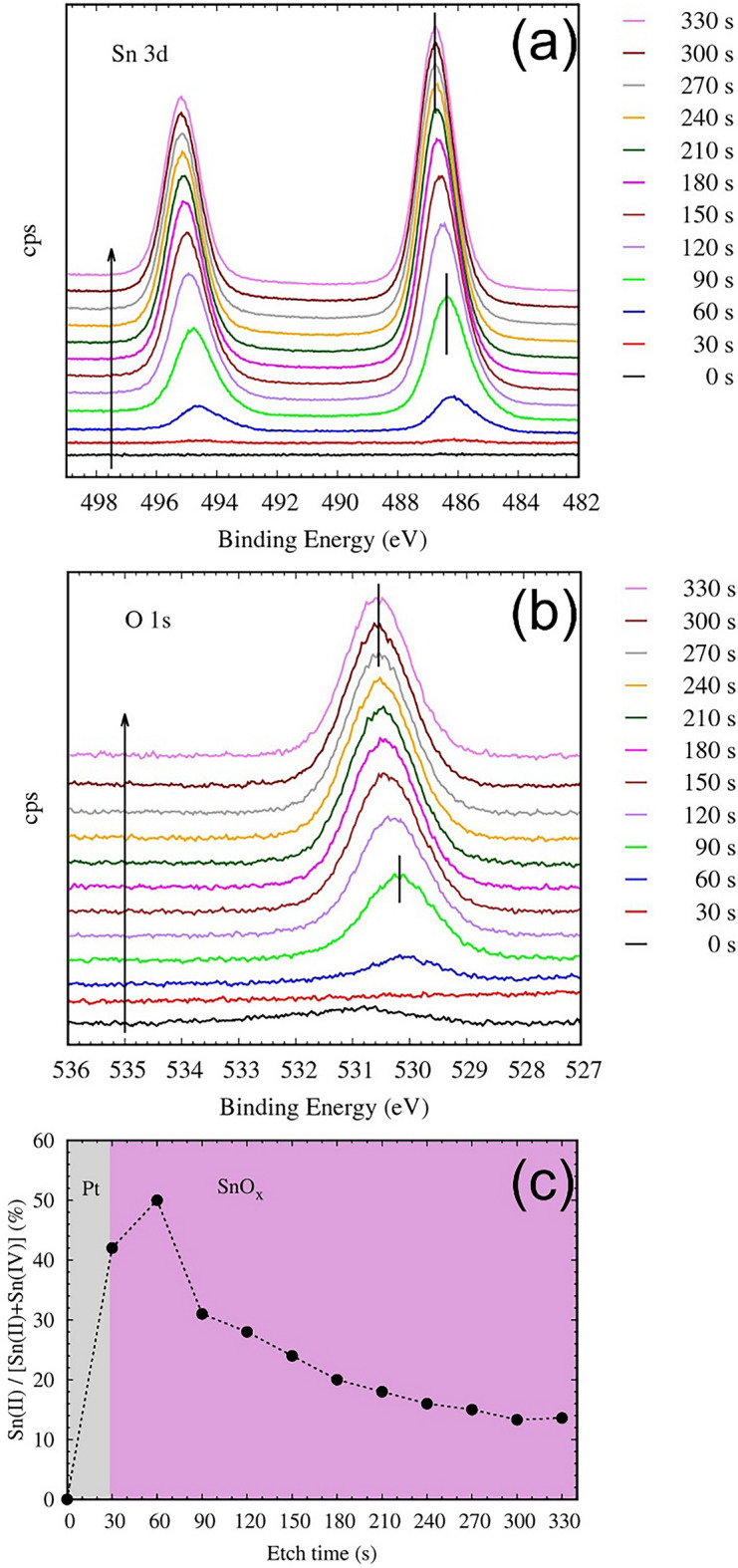
(**a**) Sn 3d and O 1 s spectra of the Pt/SnOx sample. The shift in peak positions indicate the change in the film stoichiometry as a function of the etch depth. The peak positions after 90 and 300 s of etch are marked in the figures, being (**a**) 846.38 eV and 846.77 eV for Sn 3d and (**b**) 530.18 eV and 530.55 eV for O 1 s, respectively. (**c**) the Sn(II)/[Sn(II) + Sn(IV)] ratio in the films as a function of the etch time. The Sn(II) and Sn(IV) fractions are fitted to the experimental data.

Prior to device characterization, the as-deposited films have been analyzed using XPS. According to the XPS depth profiling data the Pt/SnOx interface has a mixed SnO/SnO_2_ stoichiometry, while the film bulk consists of mainly SnO_2_. Figure 2a,b show the Sn 3d and O 1 s spectra collected in 30 s Ar + etching intervals. The change in the film stoichiometry is clearly shown as asymmetric peak shapes and a shift towards higher binding energies in the peak positions when the etching time increases^31^. This ca. 0.4 eV shift is visible in both the Sn 3d and O 1 s spectra, as shown in Fig. 2a,b. After a 60 s Ar + etch i.e., close to the Pt/SnOx interface the film contain a large fraction of Sn(II), but this fraction becomes significantly smaller in the film bulk. The larger FWHM of the Sn(II) peaks compared to the Sn(IV) indicates that the SnO species in the film are less oriented than the crystalline SnO_2_, which is consistent with the XRD data (Fig. S1, supporting information).

The ratios of Sn(II) to the total Sn (Sn(II) + Sn(IV)), as a function of the etch time, are presented in Fig. 2c, which shows that near the Pt/SnOx interface the fraction of Sn(II) species can be as high as 50%, but saturates to ca. 16% in the film bulk, however some uncertainty of the exact Sn(II) concentration remains due to the significant overlap of the Sn 3d peaks originating from the Sn(II) and Sn(IV) oxidation states, as well as the line shape used in the analysis and the introduction of etching artifacts in the spectra. Despite this uncertainty, the XPS data qualitatively confirms that a sub-stoichiometric region near the top metal electrode interface can act as a source of oxygen vacancies that enable resistive switching.

**Figure 3:**
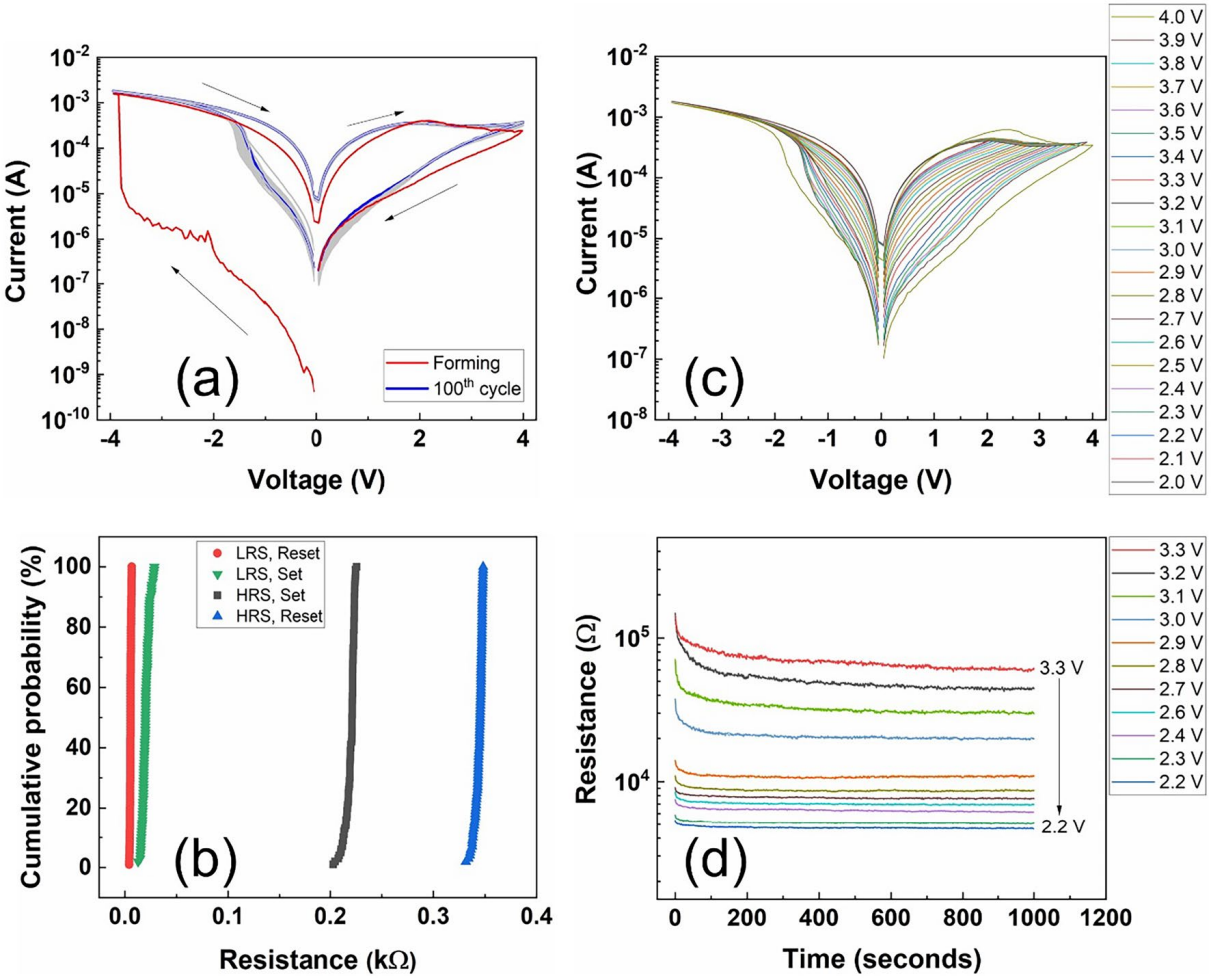
(**a**) The I–V characteristics of Ti/Pt/SnOx/Pt RRAM devices show forming (red), set (in the negative voltage region), reset (in the positive voltage region) and cycle measurements (gray lines). (**b**) The cumulative probability graphs show distribution of resistance values of LRS and HRS of both set (green and black) and reset (red and blue). The coefficient variability values of LRS-Reset, LRS-Set, HRS-Set and HRS-Reset are 0.02, 0.01, 0.16 and 0.1, respectively. (**c**) Multiple resistance states of SnO_x_ RRAM devices measured at different reset voltages (2–4 V) with a step size of 0.1 V. (**d**) The retention measurements show stable resistance states measured at different voltages for 1000 s.

The I–V characteristics of Ti/Pt/SnO_x_/Pt RRAM devices are shown in Fig. 3a. As-prepared devices have shown high resistance (in GΩ), owing to the highly oxidized state of SnO_x_ films. It is known that SnO_x_ intrinsically has oxygen vacancies and a high concentration of these can lead to n-type semiconducting behavior, which is useful for thin film transistor applications. However, in the case of RRAM devices, such high conductivity is undesirable. Therefore, in order to reduce the concentration of oxygen vacancies high O_2_/Ar ratio (2:1) has been used during sputtering process, and it led to highly resistive SnO_x_ films. Consequently, these devices required a forming step that involves applying voltage pulses with a gradual increase in amplitude. The devices switched from the pristine resistance state to the low resistance state (LRS) at ≈ − 3.9 V due to the formation of the conducting filament in the SnO_x_ film. When a positive bias of ≈ 4 V is applied, the devices switched from LRS to a high resistance state (HRS), which is also called reset. The set (HRS to LRS) occurred at a much lower voltage (≈ − 2 V) than the forming voltage and such low voltage devices are suitable for portable electronic devices (e.g., wearable electronics). The additional interesting feature of these devices is that they do not require any current compliance element to prevent the hard breakdown of the devices. The compliance-free nature is due to the high internal filament resistance of the SnO_x_ films^36^, and it is further analyzed using dynamic conductance (dI/dV vs V) graphs (Fig. S3, supporting information), which are obtained by taking the derivative of current with respect to applied voltage step (0.05 V)^37^. In the SET regime, the magnitude of dynamic conductance increases with an increase in the voltage, and before stabilizing to a higher conductance it sharply increases due to the formation of the conducting filament. In the case of RESET, the dynamic conductance increases initially with applied voltage, but decreases gradually due to the rupture of the filament and the fluctuations in the conductance might be due to the electron trapping and detrapping processes. The dynamic conductance graphs indicate that devices possess self-limiting current (intercept at zero dynamic conductance)^38^, which can be attributed to high internal resistance of the films. Next, to understand the cyclic stability of the devices, endurance measurements were performed. Figure 3a shows that the devices are quite stable over 100 cycles without any data error (no intermediate state). The fine distribution (Fig. 3b) of LRS and HRS values (at a read voltage of 0.2 V) of all these 100 cycles in both set and reset regimes further confirms the stability and uniformity of the devices. Furthermore, several devices demonstrated similar asymmetric I–V characteristics and have shown less variation in LRS and HRS values for both set and reset (Figs. S4, S5). These characteristics confirm the repeatability of prepared RRAM devices.

The gradual switching of the device from LRS to HRS in the reset regime indicates that it is an analog device with possible multiple states. Therefore, analog switching characteristics have been measured by applying the same set voltage of − 4 V, but varying reset voltages from 2 to 4 V with a step of 0.1 V (Fig. 3c). Different reset voltages lead to different states due to the accumulation of different amounts of oxygen vacancies in the filament. Next, retention measurements have been performed to find out the stability of the states, and more than 10 states are found to be stable for 1000 s (Fig. 3d). However, there is a relaxation effect (loss of retention) in the initial several seconds, which is possibly due to the diffusion of bulk oxygen vacancies and/or detrapped electrons into the filament^39^. When the system reaches a steady state, there is no significant loss in retention.

**Figure 4:**
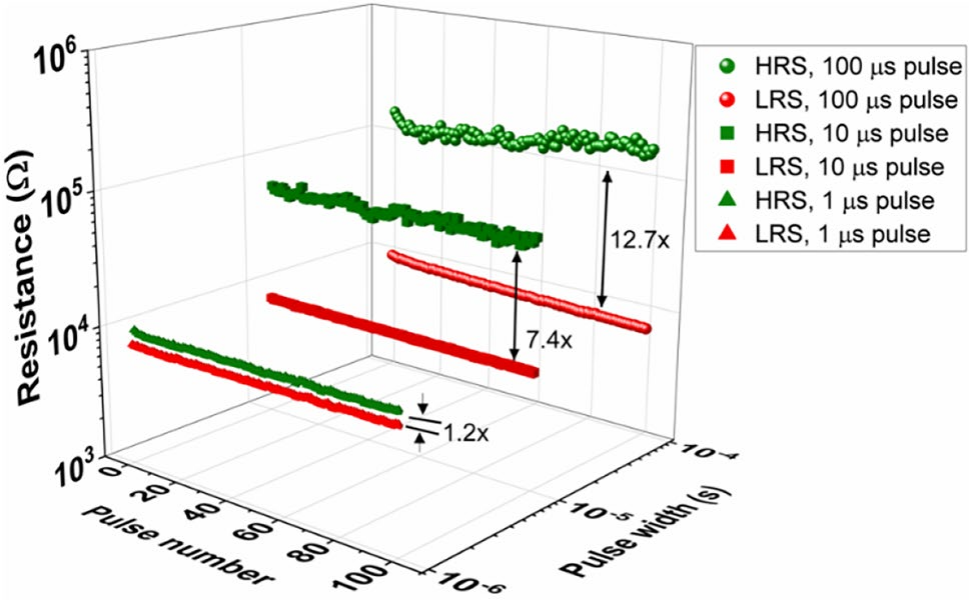
Resistive switch dynamic range of SnO_x_ RRAM devices. The resistance states depend on the pulse width for a fixed pulse amplitude (3 V). Different pulse widths lead to different HRS/LRS ratios such as 1.2, 7.4 and 12.7 for 1, 10 and 100 μs pulse widths.

Pulse width dependence of the RRAM devices has also been studied (Fig. 4) by measuring devices at different pulse widths (1, 10, and 100 μs), with the voltage from − 3 to 3 V. These measurements indicate that individual resistance states without any overlap can be obtained by varying the pulse width. Interestingly, the HRS values increased significantly with an increase in pulse width, and the average HRS values for 1,10 and 100 μs pulses are 9934 ± 169, 62,811 ± 3928 and 132,635 ± 21,150 Ω, respectively. On the other hand, the LRS values have slightly decreased with an increase in pulse width, the average LRS values for 1, 10, and 100 μs pulses are 7947 ± 119, 7868 ± 153, and 7618 ± 494 Ω, respectively. Since the applied voltage is higher than the set voltage, the devices reached to the LRS state, and the increase in the pulse width has led to small decrease in the LRS values. On the other hand, applied voltage is lower than the reset voltage, the devices have not attained lowest possible HRS and increase in the pulse width supplied enough energy to migrate the oxygen vacancies from the filament, which led to increase in the HRS values.

**Figure 5:**
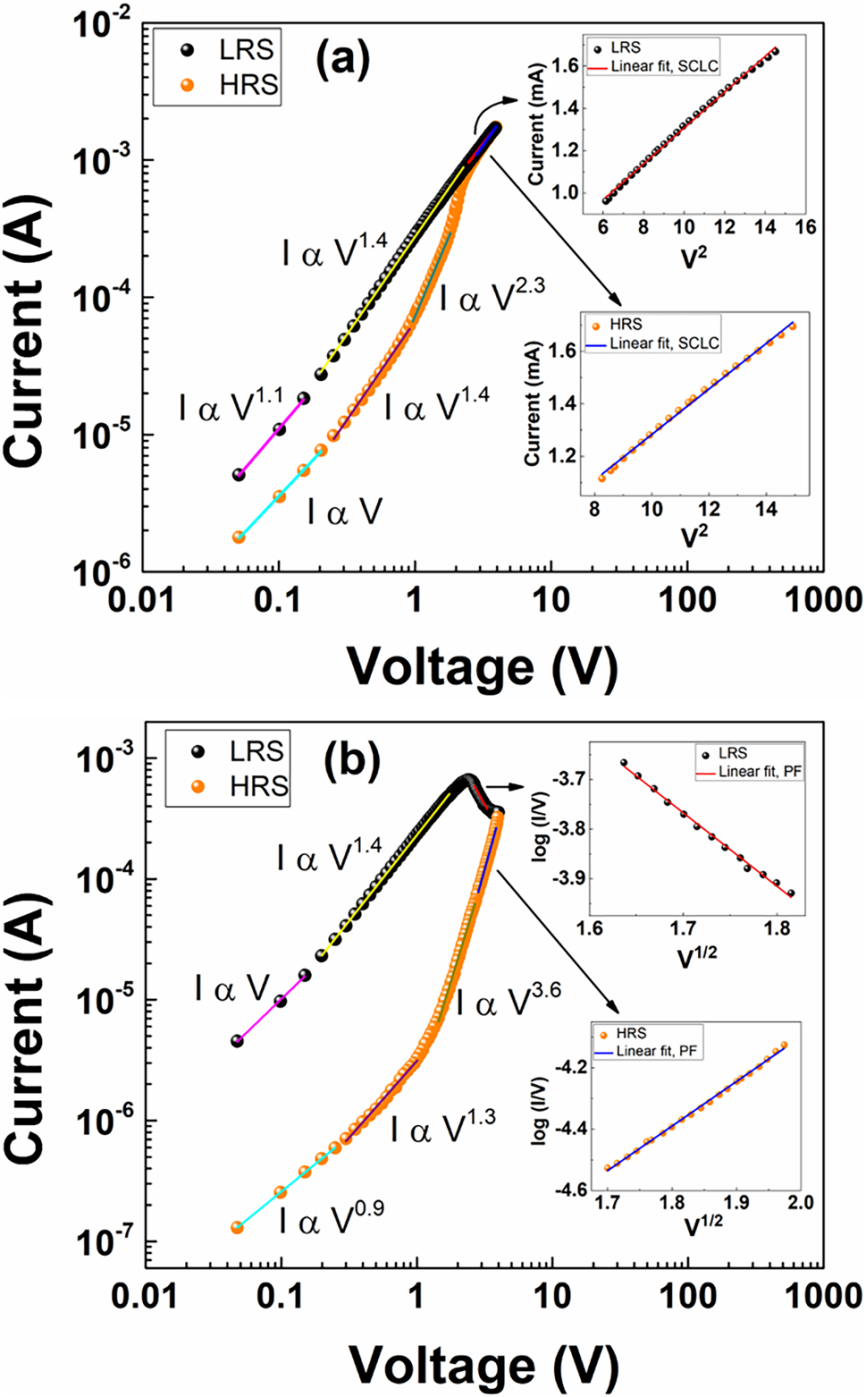
The conducting mechanism of SnO_x_ RRAM devices is obtained by fitting the set (**a**) and reset (**b**) graphs. At low voltages LRS and HRS of both set and reset fit to Ohm’s law. However, at high voltages LRS and HRS of set (inset in 5**a**) fit to SCLC mechanism. Whereas the reset graphs fit to Poole–Frenkel mechanism (inset in 5**b**).

The mechanism has been analyzed by fitting both set and reset curves with different conduction mechanism models. In case of set (Fig. 5a), both LRS and HRS shows Ohmic contact (I α V) at low voltages and as the voltage increases further, the slope changes gradually. At higher voltages, it follows Child’s law (I α V^2^), which indicates that the conduction mechanism is space charge limited conduction (SCLC). Here the conduction is due to drift of oxygen vacancies, which reduces the trap density and leads to increase in the current density sharply. On the other hand, in case of reset (Fig. 5b), Ohmic contact is present at low voltages, and slope changes with increase in the voltage. At higher voltages it follows Poole–Frenkel (P–F) conduction (log (I/V) vs. V^1/2^) mechanism, which occurs due to excitation of trapped electrons into the conduction band of tin oxide. Here, the applied electric field reduces the potential barrier, which there increases the probability of thermal excitation of electrons out of the traps^40^.Two different mechanisms (SCLC and P–F) in set and reset cause the I–V curve to be asymmetric, and the gradual reset provided the multiple resistance states, and many of these are stable. In addition, the devices do not require any external compliance current limiting devices such as resistors and/or transistors, which require additional space and reduce power efficiency. The comparison of these devices with different oxides (Table S1) indicate that endurance cycles and retention times are lower or comparable to some of the reported oxides. However, those RRAM devices require either compliance current or multiple resistance states and pulse width measurements have not been demonstrated. Therefore, the prepared SnO_x_ devices offer potential applications such as low power computing neuromorphic devices and gasistors due to the sensitive nature of SnO_x_ towards several gases.

## Conclusions

A metal-oxide-metal structure with Pt as electrodes and SnO_x_ as a switching layer was fabricated on thermally oxidized Si substrate. The compositional analysis confirms the presence of SnO_x_ consisting SnO_2_ with a small percentage of SnO. The I–V characteristics revealed non-linear resistance behaviour and the set/reset switching process occurs at a lower voltage of 2 V compared to the filament forming voltage of 4 V. This confirmed the applicability of devices in the portable electronics market. The devices exhibited endurance of 100 cycles and the retention test showed data retentivity of 1000 s for more than 10 states. By fitting the set and reset curves, the conduction mechanism was determined. At lower voltages, Ohmic conduction dominates in both the set and reset processes, whereas at higher voltages, the set and reset process were dominated by space charge conduction and by Poole–Frenkel conduction mechanisms, respectively. In conclusion, the device fabricated with SnO_x_ as switching oxide was compliance-free and exhibited analog switching characteristics, which is essential for neuromorphic computing applications.

### Supplementary Information

Supplementary Information.

## Data Availability

The datasets used and/or analysed during the current study available from the corresponding author on reasonable request.
