# Supplementary material for: Compliance-free, analog RRAM devices based on SnOx
Source: Sci Rep. 2024 Jun 19;14:14163. doi: 10.1038/s41598-024-64662-9 (PMC11187170; doi:10.1038/s41598-024-64662-9)
Supplement: Supplementary file 1 — Supplementary Information. [file 41598_2024_64662_MOESM1_ESM.docx]

**Supporting information**

1. XRD and XPS analysis of SnO_x_ films





Figure S1. XRD pattern (red) of as-deposited SnO_x_ films. The green lines indicate the standard SnO_2_ pattern (ICSD file no. 39174).





Figure S2. The valence edge spectrum of SnO_x_ films, which confirms SnO_2_ phase.

2. Dynamic conductance analysis


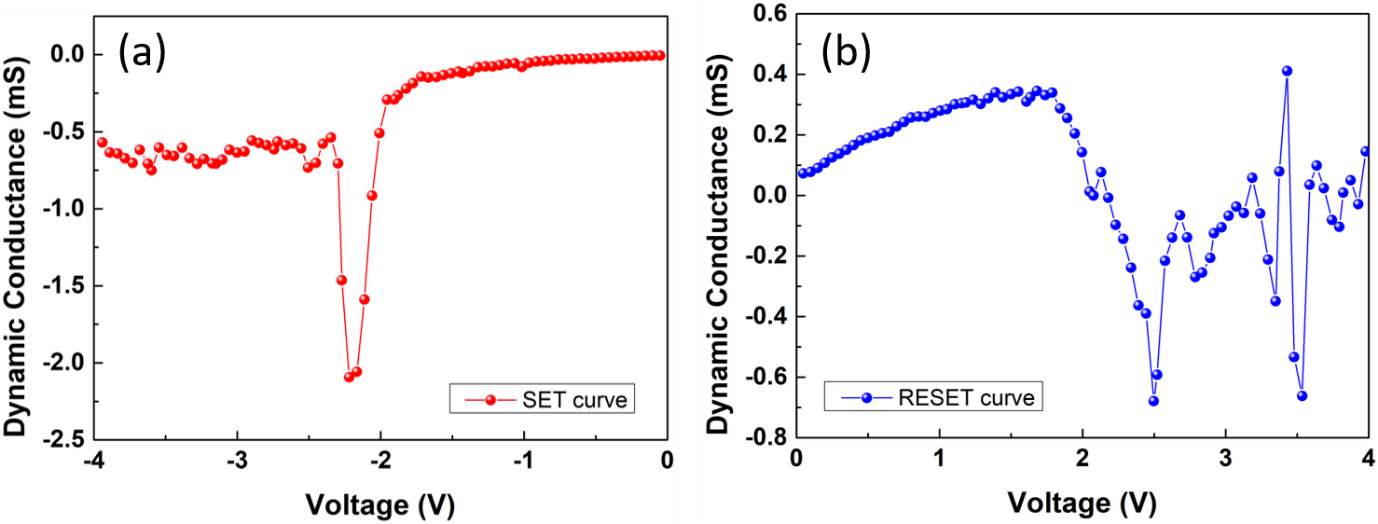


Figure S3. The dynamic conductance graphs of (a) set and (b) reset


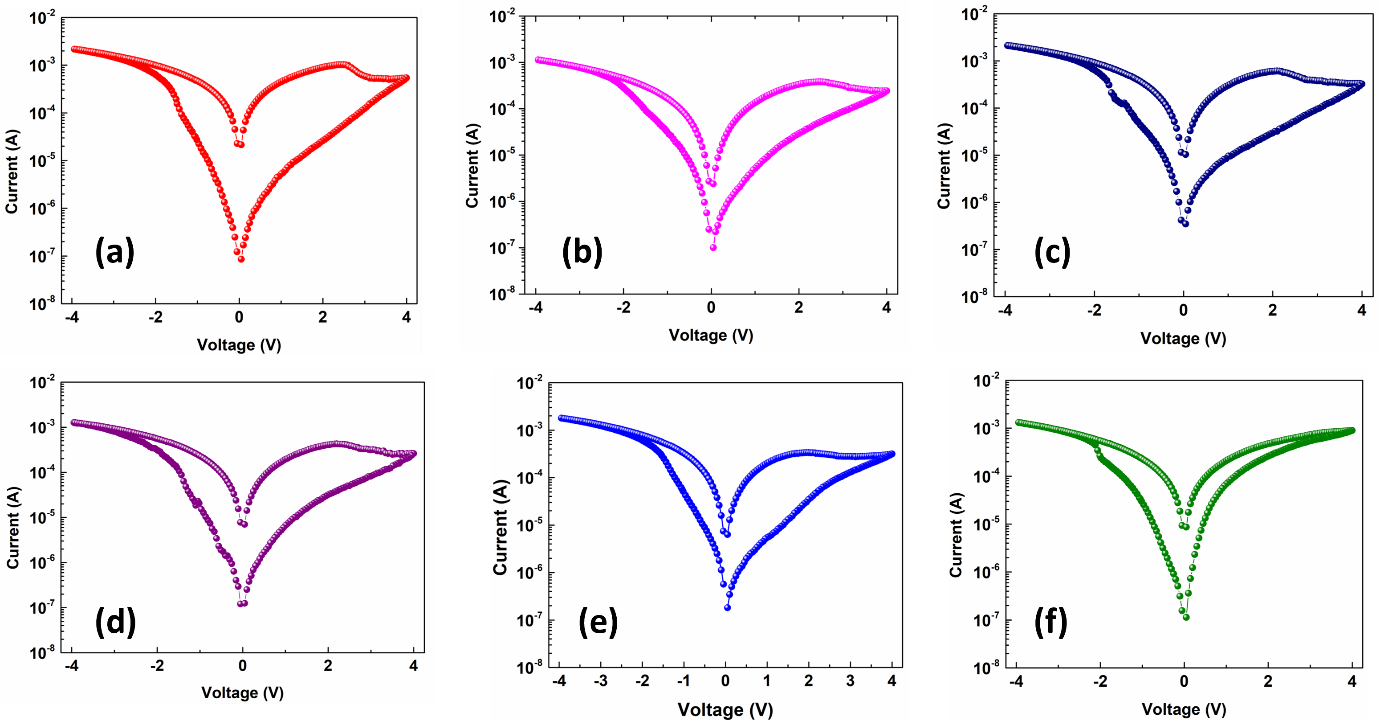


Figure S4. Resistive switching of six SnO_x_ memristor devices are shown from (a) to (f).





Figure S5. The graph shows the variation of LRS and HRS for both set and reset of 10 different SnO_x_ memristor devices.

**Table S1: This table shows different memristor device parameters of various oxides such as TiO_2_, Y_2_O_3_, SiO_2_, Ta_2_O_5_, HfO_x_ and our work.**

| **Device System** | **Method** | **Forming Voltage** | **Vset/Vreset** | **Compliance current** | **Switching**  **ratio** | **Endurance**  **cycles** | **Retention** | **Multi**  **-state** | **Ref.** |
| --- | --- | --- | --- | --- | --- | --- | --- | --- | --- |
| Ti/TiO_2-x_/Au | Reactive RF sputtering | Free |  | 1 nA | 10 | 50 |  | Yes | [1] |
| Cu/TiO_2_/Pt | DC Reactive magnetron sputtering | 0.71 V | 0.71V/-1.1 V | 10 mA |  | 10^5^ | 10^5^ s | No | [2] |
| Al/TiO_2_/Al | RF Magnetron reactive sputtering | 2 V |  | 300 µA | 2 | 100 | 10^3^ s | No | [3] |
| Pt/TiO_x_/TiO_2_/  TiO_x_/Pt | Magnetron sputtering | Free | +0.65 V |  | <10 | 10^5^ | 10^6^ s | No | [4] |
| Al/Y_2_O_3_/GZO | Dual ion beam sputtering (DIBS) | Free | 1.8V / - 1.93 V | 20µA | >200 | 7x10^5^ | 1.5 × 10^5^ s | No | [5] |
| Al/Y_2_O_3_/GZO | DIBS |  | 2.72V/ -2*.*34 V | 300 µA |  | 90 |  | No | [6] |
| Pt/Y_2_O_3_/Pt | RF Magnetron reactive  sputtering | -1.6V | 2.68V/ -1.50 V | 1 mA | >10 | 220 |  | Yes | [7] |
| Ag/SiO_2_/Pt NPs | RF Magnetron sputtering | Free | 180-200 mV | 100 µA | 10^6^ | 300 | 10^5^ s | No | [8] |
| Ag/SiO_2_/VO_x_/TiN | RF Magnetron Sputtering | Free | 180-200mV/ - 100mV | Self | 10^5^ | 300 | 10^5^ s | No | [8] |
| Ag/ASTO/SiO_2_/p-Si | RF Magnetron  Sputtering | 2.8 V | 2.8V/-4.5V | 10 mA | 10^2^ | 4 x 10^2^ | 10^4^ s | No | [9] |
| Ti/Ta_2_O_5_/Pt | RF Magnetron Sputtering | 3 V | 1.4V/-0.9V | 5 mA |  | 200 | 10^4^ s | Yes | [10] |
| Ni/Ta_2_O_5_/p++-Si | RF Magnetron Sputtering | 10 V | 5.1V/-2.8-3.8V | 10 mA | 10^3^ | 135 | 10^4^ s | No | [11] |
| W/Hf/TaO_y_/TaO_x_/Pt | RF Magnetron Sputtering | 5.5 V | 2.6V/-2.8V |  | 20 | 10^8^ | 10^4^ s | Yes | [12] |
| Ag/Ta_2_O_5_/Pt | RF Sputtering | 2.5 V | 0.5V/-0.6V | 20 mA | 10^3^ |  | 10^4^ s | No | [13] |
| Cu/HfO_2_/W | RF sputtering | 1.25 V | 0.6 V/ -0.6 V | 10 µA |  | 500 | 10^4^ s | No | [14] |
| V/HfO_2_/TiN | RF sputtering | 5.1 V |  | 100µA | 10^3^ | 10^7^ | 2 x 10^4^ s | No | [15] |
| TiN/Ti/HfO_2_/Pt | ALD | 2.65 V | 1.2V/-2 V | 10 µA |  | 5 × 10^5^ | 3.2x10^8^ s | Yes | [16] |
| Ti/Pt/SnO_x_/Pt | Magnetron sputtering | -3.9 V | -2 V/ 4 V | Self |  | >100 | >10^3^ s | Yes | This work |

**References:**

1. Tsigkourakos, M., Bousoulas, P., Aslanidis, V., Skotadis, E. & Tsoukalas, D. Ultra-Low Power Multilevel Switching with Enhanced Uniformity in Forming Free TiO2−x-Based RRAM with Embedded Pt Nanocrystals. *Phys. Status Solidi Appl. Mater. Sci.* **214,** (2017).

2. Rathore, B. P. S., Prakash, R. & Kaur, D. Effect of AlN layer on the resistive switching properties of TiO2 based ReRAM memory devices. *Curr. Appl. Phys.* **18,** 102–106 (2018).

3. Gul, F. Carrier transport mechanism and bipolar resistive switching behavior of a nano-scale thin film TiO2 memristor. *Ceram. Int.* **44,** 11417–11423 (2018).

4. Srivastava, S., Thomas, J. P., Guan, X. & Leung, K. T. Induced Complementary Resistive Switching in Forming-Free TiOx/TiO2/TiOxMemristors. *ACS Appl. Mater. Interfaces* (2021). doi:10.1021/acsami.1c09775

5. Kumar, S., Das, M., Htay, M. T., Sriram, S. & Mukherjee, S. Electroforming-Free Y2O3Memristive Crossbar Array with Low Variability. *ACS Appl. Electron. Mater.* **4,** 3080–3087 (2022).

6. Kumar, S., Agarwal, A. & Mukherjee, S. Electrical Performance of Large-Area Y2O3Memristive Crossbar Array With Ultralow C2C Variability. *IEEE Trans. Electron Devices* **69,** 3660–3666 (2022).

7. Guo, Z. *et al.* Tuning oxygen vacancies and resistive switching behaviors in amorphous Y2O3 film-based memories. *J. Alloys Compd.* **923,** (2022).

8. Bousoulas, P., Sakellaropoulos, D. & Tsoukalas, D. Tuning the analog synaptic properties of forming free SiO2memristors by material engineering. *Appl. Phys. Lett.* **118,** (2021).

9. Ilyas, N. *et al.* A Modified SiO2-Based Memristor with Reliable Switching and Multifunctional Synaptic Behaviors. *J. Phys. Chem. Lett.* **13,** 884–893 (2022).

10. Abbas, Y. *et al.* Structural engineering of tantalum oxide based memristor and its electrical switching responses using rapid thermal annealing. *J. Alloys Compd.* **759,** 44–51 (2018).

11. Ryu, J. H. *et al.* Filamentary and interface switching of CMOS-compatible Ta2O5 memristor for non-volatile memory and synaptic devices. *Appl. Surf. Sci.* **529,** (2020).

12. Alamgir, Z., Beckmann, K., Holt, J. & Cady, N. C. Pulse width and height modulation for multi-level resistance in bi-layer TaOx based RRAM. *Appl. Phys. Lett.* **111,** (2017).

13. Chang, C. F. *et al.* Direct Observation of Dual-Filament Switching Behaviors in Ta2O5-Based Memristors. *Small* **13,** (2017).

14. Das, O. P. & Pandey, S. K. Exploring the Physical Properties Related to Resistive Switching Events in HfO 2 -Based RRAM Devices with an Analytical Framework . *ACS Appl. Electron. Mater.* **5,** 327–334 (2023).

15. Lin, C. Y. *et al.* Attaining resistive switching characteristics and selector properties by varying forming polarities in a single HfO2-based RRAM device with a vanadium electrode. *Nanoscale* **9,** 8586–8590 (2017).

16. Sun, J., Tan, J. B. & Chen, T. HfOx-Based RRAM Device with Sandwich-Like Electrode for Thermal Budget Requirement. *IEEE Trans. Electron Devices* **67,** 4193–4200 (2020).
